# Harnessing Whole Genome Polygenic Risk Scores to Stratify Individuals Based on Cardiometabolic Risk Factors and Biomarkers at Age 10 in the Lifecourse—Brief Report

**DOI:** 10.1161/ATVBAHA.121.316650

**Published:** 2022-01-20

**Authors:** Tom G. Richardson, Katie O’Nunain, Caroline L. Relton, George Davey Smith

**Affiliations:** Bristol Medical School (T.G.R., K.O., C.L.R., G.D.S.), University of Bristol, United Kingdom.; MRC Integrative Epidemiology Unit (IEU), Population Health Sciences, Bristol Medical School (T.G.R., C.L.R., G.D.S.), University of Bristol, United Kingdom.; Novo Nordisk Research Centre, Headington, Oxford, United Kingdom (T.G.R.).

**Keywords:** ALSPAC, biomarkers, lipids, polygenic risk scores, risk factors

## Abstract

In this study, we investigated the capability of polygenic risk scores to stratify a cohort of young individuals into risk deciles based on 10 different cardiovascular traits and circulating biomarkers.

**Methods:**

We first conducted large-scale genome-wide association studies using data on adults (mean age 56.5 years) enrolled in the UK Biobank study (n=393 193 to n=461 460). Traits and biomarkers analyzed were body mass index, systolic blood pressure, diastolic blood pressure, high-density lipoprotein cholesterol, low-density lipoprotein cholesterol, triglycerides, apolipoprotein B, apolipoprotein A-I, C-reactive protein and vitamin D. Findings were then leveraged to build whole genome polygenic risk scores in participants from the Avon Longitudinal Study of Parents and Children (mean age, 9.9 years) which were used to stratify this cohort into deciles in turn and analyzed against their respective traits.

**Results:**

For each of the 10 different traits assessed, we found strong evidence of an incremental trend across deciles (all *P*<0.0001). Large differences were identified when comparing top and bottom deciles; for example, using the apolipoprotein B polygenic risk scores there was a mean difference of 13.2 mg/dL for this established risk factor of coronary heart disease in later life.

**Conclusions:**

Although the use of polygenic prediction in a clinical setting may currently be premature, our findings suggest they are becoming increasingly powerful as a means of predicting complex trait variation at an early stage in the lifecourse.

## Abstract 2

Supplemental Digital Content is available in the text.

**Highlights**

- Using genetic data from up to 461 460 adults from the UK Biobank study, we derived weights to construct whole genome polygenic risk scores for 10 different cardiometabolic traits and biomarkers.
- We then built polygenic risk scores using data from a cohort of young individuals enrolled in the Avon Longitudinal Study of Parents and Children (mean age 9.9 years) who additionally had measures for each of the 10 different traits.
- Each of the 10 different polygenic risk scores were found to be strong genetic predictors capable of accurately stratifying participants into risk deciles during this early stage in the lifecourse.

Polygenic risk scores (PRS) involve the aggregation of genetic variants scattered throughout the human genome to index an individual’s genetic risk of disease.^1^ Their use in applied research has become increasingly popular in recent years, although their diagnostic capabilities in clinical settings remains a contentious point of discussion.^2^ Nevertheless, their utility in terms of stratifying cohorts of participants into high and low risk groups based entirely on their genetic variation continues to improve. This is predominantly due to samples sizes for genome-wide association studies (GWAS) continuing to grow in scale, which are conventionally used to identify weights for PRS.^3^

As an individual’s inherited genetic variants are typically fixed at conception, one of the major strengths of PRS is that they can be applied to identify participants at elevated risk of disease at an early stage in the lifecourse. A recent study by Khera et al explored this by harnessing a large number of genetic variants from across the human genome and constructing PRS in a longitudinal cohort of young individuals from the ALSPAC (Avon Longitudinal Study of Parents and Children).^4–6^ Their PRS was capable of accurately stratifying participants into high and low risk groups based on measures of weight during childhood, for example identifying a difference of 3.5 kg between the top and bottom deciles of participants by age 8 years (*P*<0.0001).

In this study, we conducted large-scale GWAS of 10 different cardiometabolic risk factors and circulating biomarkers based on an adult population (mean age: 56.5 years) enrolled in the UKB (UK Biobank) study.^7^ Findings from these analyses were then leveraged to derive whole genome PRS within the ALSPAC cohort to investigate the proficiency of PRS to stratify individuals during childhood (mean age, 9.9 years) into low and high risk groups based on their measures of each of these 10 different traits.

## Materials and Methods

Because of the sensitive nature of the data collected for this study, requests to access the dataset from qualified researchers trained in human subject confidentiality protocols may be sent to the UK Biobank at https://www.ukbiobank.ac.uk/enable-your-research/apply-for-access and ALSPAC at http://www.bristol.ac.uk/alspac/researchers/access/.

### GWAS in the UK Biobank

We conducted 10 GWAS in the UKB study on the following traits; body mass index (field No. 21001), systolic blood pressure (field No. 4080), diastolic blood pressure (field No. 4079), HDL-C (high-density lipoprotein cholesterol; field No. 30760), LDL-C (low-density lipoprotein cholesterol; field No. 30780), triglycerides (field No. 30870), apolipoprotein B (field No. 30640), apolipoprotein A-I (field No. 30630), C-reactive protein (field No. 30710), and vitamin D (field No. 30890; Table S1). The analysis protocol for these GWAS has been described in more details previously.^8^ Briefly, we excluded UKB participants on non-European descent based on K-means clustering (K=4) along with individuals with withdrawn consent, mismatch between genetic and reported sex and putative sex chromosome aneuploidy. GWAS were then conducted using the BOLT-LMM software which accounts for population structure and cryptic relatedness in UKB using a linear mixed model.^9^ Analyses were additionally adjusted for age and sex with final sample sizes ranging between n=393 193 and n=461 460.

### The Avon Longitudinal Study of Parents and Children

ALSPAC is a population-based cohort investigating genetic and environmental factors that affect the health and development of children. The study methods are described in detail elsewhere.^5,6^ In brief, 14 541 pregnant women residents in the former region of Avon, United Kingdom, with an expected delivery date between April 1, 1991, and December 31, 1992, were eligible to take part in ALSPAC. Detailed phenotypic information, biological samples, and genetic data which have been collected from the ALSPAC participants are available through a searchable data dictionary (http://www.bris.ac.uk/alspac/researchers/our-data/). Written informed consent was obtained for all study participants. Ethical approval for this study was obtained from the ALSPAC Ethics and Law Committee and the Local Research Ethics Committees.

We identified the same 10 traits as listed above in ALSPAC using data obtained from participants enrolled at mean age=9.9 years old clinic (range=8.9 to 11.5 years old). Trait characteristics in ALSPAC can be found in Table S2.

### Statistical Analysis

We pruned the full list of genetic variants from GWAS results obtained from UKB analyses using linkage disequilibrium clumping. Our criteria was based on variants with an *r*^2^<0.1 and window distance of 1000 kbs using a previously derived reference panel of 10 000 random UKB European participants.^10^ Next, we built PRS for each of the 10 cardiometabolic traits and circulating biomarkers using data from ALSPAC participants by summing trait increasing alleles weighted by their GWAS effect estimates. Linkage disequilibrium clumping and PRS construction were all performed using the software PLINK v2.0.^11^

We applied linear regression adjusting for age and sex to investigate the association between each whole genome PRS in ALSPAC in turn with their corresponding cardiometabolic trait or circulating biomarker. Analyses were repeated with additional adjustment for the top 10 genetic principal components as a sensitivity analysis, although we did not envisage that population stratification would influence findings given that all participants from the ALSPAC cohort were based in the country of Avon in the United Kingdom. Log transformations were conducted to ensure that traits were normally distributed. Next, we compared the proportion of variance explained between baseline models which just included age and sex with those additionally including the whole genome PRS.

Lastly, PRS were used to stratify the ALSPAC population into deciles and linear regression was applied again to investigate evidence of a linear trend across groups for each trait in turn. As a sensitivity analysis, we also compared the performance of the apolipoprotein B PRS in stratifying ALSPAC participants into deciles based on their measure of non-HDL cholesterol. This was derived by subtracting ALSPAC individuals’ measures of HDL cholesterol from their measures of total cholesterol.

## Results

We found strong evidence of association between each of the 10 whole genome PRS and their corresponding traits measured in predominantly prepubertal individuals enrolled in the ALSPAC study (Table S3). As expected, repeating analyses with additional adjustment for the top 10 genetic principal components made negligible differences to results (Table S4). Furthermore, the proportion of variance explained increased dramatically by including PRS into baseline models (Table S5), with the largest change being identified for HDL cholesterol (*r*^2^=0.095). Additionally, we observed clear incremental trends across deciles after stratifying the ALSPAC sample according to each of the 10 whole genome PRS (Figure), with large mean differences found between top and bottom deciles. For example, in the analysis regarding apolipoprotein B, a risk factor for coronary artery disease in later life,^8,12^ the mean measure among participants allocated to the top decile was 65.4 mg/dL, which was markedly different to the mean level of those grouped in the bottom decile (52.6 mg/dL). A strong linear trend was additionally found across deciles of non-HDL cholesterol using the apolipoprotein B PRS (*P*=3×10^−64^; Figure S1). The weakest evidence of a linear trend using these PRS was for C-reactive protein (*P*=7×10^−05^), which we postulate may be due to the factors contributing to GWAS associations identified in a population of adults having less influence during childhood. All other results from this analysis can be found in Table S6.

**Figure.**
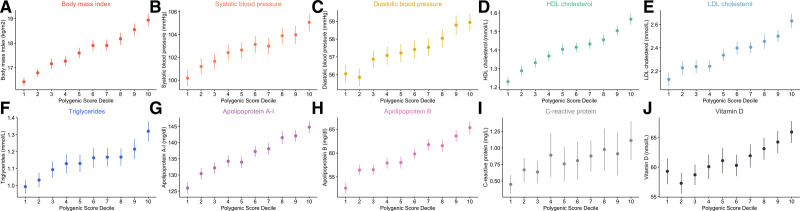
**Error plots illustrating the mean measurement and 95% CIs within deciles as determined using whole genome polygenic risk scores (PRS) applied within the ALSPAC (Avon Longitudinal Study of Parents and Children) cohort.** Each PRS was weighted using on findings from genome-wide association studies on their corresponding traits undertaken in populations of adults enrolled in the UK Biobank study. HDL indicates high-density lipoprotein; and LDL, low-density lipoprotein.

## Discussion

The findings of this work provide compelling evidence supporting the power of whole genome PRS in helping prioritize individuals with elevated levels of cardiometabolic traits and biomarkers during early life. This is likely due in no small part to recent large-scale GWAS sample sizes, which are anticipated to grow exponentially over the forthcoming years. Ultimately, the future of polygenic prediction may exist when being applied in conjunction with nongenetic risk factors, such as molecular traits. For example, integrating PRS with data on DNA methylation, an epigenetic marker which unlike germline genetic variation may substantially vary throughout the lifecourse, may yield additive benefit to disease prediction.^13^ Likewise, information on family history may further improve polygenic prediction, with a recent study suggesting this may be particularly valuable for endeavours conducted in diverse populations of non-European ancestry.^14^ Future methodology in this space requires careful consideration regarding the most appropriate manner to integrate these types of data, particularly as combining them under the assumption of orthogonality (ie, whether 2 variables lie perpendicular to one another and, therefore, contribute independent information) is likely to inflate the predictive performance of these models.^15^ This is particularly attractive, given that the PRS leveraged in this study typically explained a relatively small proportion of variance in their corresponding traits, which has been reported by previous investigations of PRS.^16^ Furthermore, although GWAS performed in cohorts of adults are typically available in far larger sample sizes compared with those undertaken in young populations,^17^ it has been demonstrated recently that PRS weighted by GWAS estimates derived using childhood-based measures may be optimal in predicting complex traits in early life.^18^

## Conclusions

While the potential use of PRS in a clinical setting remains premature, the findings of our study suggest that they may provide future merit in terms of considering interventions at an early stage in the lifecourse.

## Article Information

### Acknowledgments

We are extremely grateful to all the families who took part in this study, the midwives for their help in recruiting them and the whole ALSPAC (Avon Longitudinal Study of Parents and Children) team, which includes interviewers, computer and laboratory technicians, clerical workers, research scientists, volunteers, managers, receptionists, and nurses. The UK Medical Research Council and Wellcome (Grant ref: 217065/Z/19/Z) and the University of Bristol provide core support for ALSPAC. Genetic data were generated by Sample Logistics and Genotyping Facilities at the Wellcome Trust Sanger Institute and LabCorp (Laboratory Corporation of America) using support from 23andMe. This research was conducted at the National Institute for Health Research Biomedical Research Centre at the University Hospitals Bristol NHS Foundation Trust and the University of Bristol. The views expressed in this publication are those of the author(s) and not necessarily those of the NHS, the National Institute for Health Research or the Department of Health. This publication is the work of the authors and T.G. Richardson will serve as guarantor for the contents of this article. All individual level data analyzed in this study can be accessed via an approved application to ALSPAC (http://www.bristol.ac.uk/alspac/researchers/access/) and the UK Biobank study (https://www.ukbiobank.ac.uk/enable-your-research/apply-for-access). Genome-wide association studies were conducted in the UK Biobank under application #15825. Written informed consent was obtained for all study participants. Ethical approval for this study was obtained from the ALSPAC Ethics and Law Committee and the Local Research Ethics Committees.

### Sources of Funding

This work was supported by the Integrative Epidemiology Unit which receives funding from the UK Medical Research Council and the University of Bristol (MC_UU_00011/1, MC_UU_00011/5).

### Disclosures

T.G. Richardson is employed part-time by Novo Nordisk outside of this work. The other authors report no conflicts.

### Supplemental Materials

Tables S1–S6

Figure S1

## Supplementary Material


