# Supplementary material for: Harnessing Whole Genome Polygenic Risk Scores to Stratify Individuals Based on Cardiometabolic Risk Factors and Biomarkers at Age 10 in the Lifecourse—Brief Report
Source: Arterioscler Thromb Vasc Biol. 2022 Jan 20;42(3):362–5. doi: 10.1161/ATVBAHA.121.316650 (PMC8860202; doi:10.1161/ATVBAHA.121.316650)
Supplement: Supplementary file 1 [file atv-42-362-s001.pdf]

SUPPLEMENTAL MATERIALS

Harnessing whole genome polygenic risk scores to stratify individuals based on cardiometabolic risk factors and biomarkers at age 10 in the lifecourse

Tom G Richardson[1,2,3,*], Katie O'Nunain[1], Caroline L Relton[1,2], George Davey Smith[1,2]

[1] *Bristol Medical School, University of Bristol, Oakfield House, Oakfield Grove, Bristol, BS8 2BN, United Kingdom*

[2] *MRC Integrative Epidemiology Unit (IEU), Population Health Sciences, Bristol Medical School, University of Bristol, Oakfield House, Oakfield Grove, Bristol, BS8 2BN, United Kingdom*

[3] *Novo Nordisk Research Centre, Headington, Oxford, OX3 7FZ, United Kingdom*

Table S1: UK Biobank traits

Trait	UK Biobank ID	Sample size	Link
Body mass index	21001	461460	https://biobank.ndph.ox.ac.uk/showcase/field.cgi?id=21001
Systolic blood pressure	4080	436419	https://biobank.ndph.ox.ac.uk/showcase/field.cgi?id=4080
Diastolic blood pressure	4079	436424	https://biobank.ndph.ox.ac.uk/showcase/field.cgi?id=4079
High density lipoprotein	30760	403943	https://biobank.ndph.ox.ac.uk/showcase/field.cgi?id=30760
Low density lipoprotein	30780	440546	https://biobank.ndph.ox.ac.uk/showcase/field.cgi?id=30780
Triglycerides	30870	441016	https://biobank.ndph.ox.ac.uk/showcase/field.cgi?id=30870
Apolipoprotein A-I	30630	393193	https://biobank.ndph.ox.ac.uk/showcase/field.cgi?id=30630
Apolipoprotein B	30640	439214	https://biobank.ndph.ox.ac.uk/showcase/field.cgi?id=30640
C-reactive protein	30710	438131	https://biobank.ndph.ox.ac.uk/showcase/field.cgi?id=30710
Vitamin D	30890	419718	https://biobank.ndph.ox.ac.uk/showcase/field.cgi?id=30890

Table S2: Raw trait characteristics in the ALSPAC cohort prior to data cleaning

Trait	Sample size	Mean	SD
Body mass index (kg/m2)	6219	17.68	2.84
Systolic blood pressure (mmHg)	6201	102.72	9.26
Diastolic blood pressure (mmHg)	6204	57.38	6.36
High density lipoprotein (mmol/L)	4553	1.4	0.31
Low density lipoprotein (mmol/L)	4011	2.35	0.61
Triglycerides (mmol/L)	4011	1.14	0.57
Apolipoprotein A-I (g/L)	4011	136.13	19.97
Apolipoprotein B (g/L)	4011	59.27	13.27
C-reactive protein (mg/L)	4011	0.71	1.96
Vitamin D (nmol/L)	4011	61.32	19.88
non-HDL cholesterol (mmol/L)	4011	2.87	0.67

Table S3: Linear regression results adjusted for age and sex

Trait	Beta	SE	P
Body mass index	0.040	0.002	5.54E-93
Systolic blood pressure	0.014	0.001	8.86E-30
Diastolic blood pressure	0.017	0.001	5.72E-31
High density lipoprotein	0.068	0.003	2.29E-90
Low density lipoprotein	0.061	0.004	1.21E-51
Triglycerides	0.072	0.007	3.63E-27
Apolipoprotein A-I	0.041	0.002	3.80E-73
Apolipoprotein B	0.063	0.003	9.83E-73
C-reactive protein	0.214	0.018	1.63E-31
Vitamin D	0.044	0.005	2.98E-16

Table S4: Linear regression results adjusted for age, sex and the top 10 principal components

Trait	Beta	SE	P
Body mass index	0.041	0.002	2.06E-87
Systolic blood pressure	0.014	0.001	1.28E-27
Diastolic blood pressure	0.017	0.002	2.95E-27
High density lipoprotein	0.061	0.004	1.22E-45
Low density lipoprotein	0.069	0.003	6.82E-84
Triglycerides	0.077	0.007	6.95E-28
Apolipoprotein A-I	0.041	0.002	3.65E-65
Apolipoprotein B	0.063	0.004	5.13E-65
C-reactive protein	0.045	0.006	4.39E-16
Vitamin D	0.214	0.019	4.81E-29

Table S5: Proportion of variance explained by baseline and polygenic risk score (PRS) models

	r2 values		
**Trait**	**age + sex**	**PRS + age + sex**	**Difference**
Body mass index	0.02	0.09	0.07
Systolic blood pressure	0.01	0.03	0.02
Diastolic blood pressure	0.00	0.03	0.02
High density lipoprotein	0.01	0.11	0.10
Low density lipoprotein	0.03	0.08	0.05
Triglycerides	0.01	0.04	0.03
Apolipoprotein A-I	0.02	0.10	0.08
Apolipoprotein B	0.03	0.11	0.08
C-reactive protein	0.04	0.07	0.03
Vitamin D	0.01	0.03	0.02

Table S6: P-values for trend across polygenic risk scores determined deciles

Trait	P-value for trend
Body mass index	8.00E-96
Systolic blood pressure	3.00E-30
Diastolic blood pressure	9.00E-32
HDL cholesterol	1.00E-98
LDL cholesterol	5.00E-55
Triglycerides	9.00E-23
Apolipoprotein A-I	3.00E-78
Apolipoprotein B	1.00E-75
C-reactive protein	7.00E-05

Vitamin D	1.00E-14
non-HDL cholesterol	3.00E-64

Figure S1: An error plot demonstrating a linear trend across deciles of non-HDL cholesterol using the apolipoprotein B polygenic risk score in ALSPAC

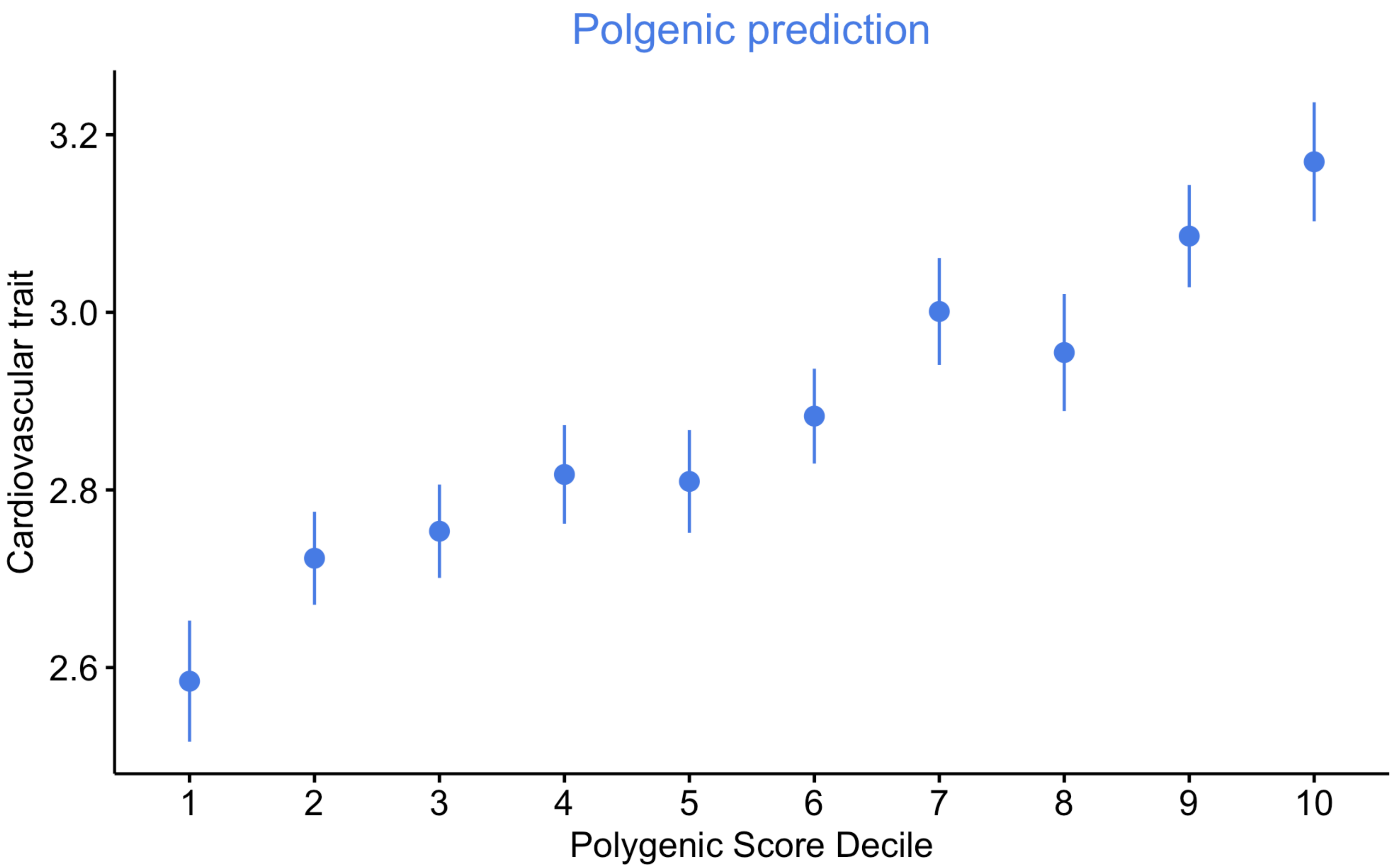

Major Resource Table

Animals (in vivo studies)

Species	Vendor or Source	Background Strain	Sex	Persistent ID / URL
NA				

Genetically Modified Animals

	Species	Vendor or Source	Background Strain	Other Information	Persistent ID / URL
**Parent - Male**	NA				
**Parent - Female**	NA				

Antibodies

Target antigen	Vendor or Source	Catalog #	Working concentration	Lot # (preferred but not required)	Persistent ID / URL
NA					

DNA/cDNA Clones

Clone Name	Sequence	Source / Repository	Persistent ID / URL
NA			

Cultured Cells7

Name	Vendor or Source	Sex (F, M, or unknown)	Persistent ID / URL
NA			

Data & Code Availability

Description	Source / Repository	Persistent ID / URL
Data from the ALSAPC cohort is available upon an approved application.	ALSPAC	http://www.bristol.ac.uk/alspac/researchers/access/

Data from the UK Biobank cohort is available upon an approved application.	UK Biobank	https://www.ukbiobank.ac.uk/enable-your-research/apply-for-access

Other

Description	Source / Repository	Persistent ID / URL
NA		
