# Supplementary figures and images for: Harnessing Whole Genome Polygenic Risk Scores to Stratify Individuals Based on Cardiometabolic Risk Factors and Biomarkers at Age 10 in the Lifecourse—Brief Report
Source: Arterioscler Thromb Vasc Biol. 2022 Jan 20;42(3):362–5. doi: 10.1161/ATVBAHA.121.316650 (PMC8860202; doi:10.1161/ATVBAHA.121.316650)

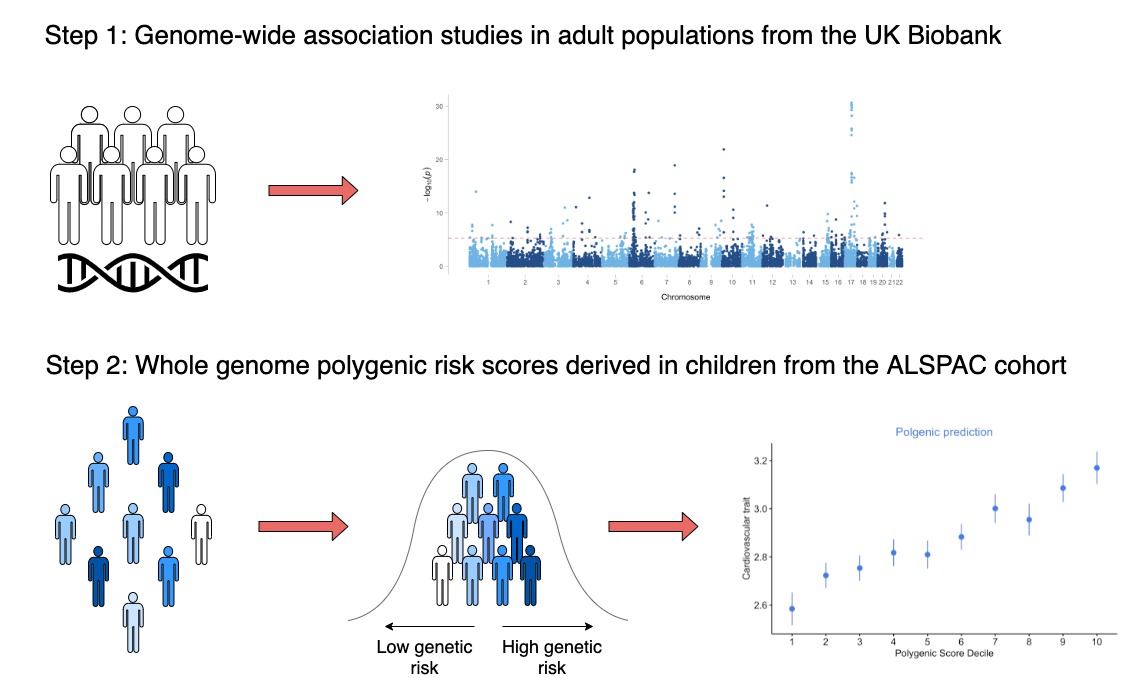

Supplement: Supplementary file 2 [file atv-42-362-s002.jpg]
